# Bioaccumulation Experiments in Mussels Contaminated with the Food-Borne Pathogen *Arcobacter butzleri*: Preliminary Data for Risk Assessment

**DOI:** 10.1155/2013/153419

**Published:** 2013-09-12

**Authors:** Donatella Ottaviani, Serena Chierichetti, Elena Rocchegiani, Chiara Bartolini, Laura Masini, Sabrina Santarelli, Francesca Leoni

**Affiliations:** Istituto Zooprofilattico Umbria e Marche, Sezione di Ancona, Laboratorio Nazionale di Riferimento (LNR) Contaminazioni Batteriologiche Molluschi Bivalvi Vivi, Via Cupa di Posatora 3, 60126 Ancona, Italy

## Abstract

The aim of this study was to evaluate, at a laboratory scale, the ability of this microorganism to grow in seawater and bioaccumulate in mussels (*Mytilus galloprovincialis*) maintained in constantly aerated tanks, containing twenty litres of artificial seawater. Three concentrations of *A. butzleri* LMG 10828^T^ were tested (about 5 × 10^6^ CFU/mL, 5 × 10^4^ CFU/mL, and 5 × 10^2^ CFU/mL). Following contamination, enumeration of *A. butzleri* was performed from water and mussels each day, for up to 96 h. Three contamination experiments with artificial seawater in absence of mussels were also performed in the same manner. In the experiments with mussels, *A. butzleri* declined in water of approximately 1 log every 24 h from the contamination. In artificial seawater without mussels the concentration of *A. butzleri* remained on the same logarithmic level in the first 48 h and then decreased of about 1 log every 24 hours. In mussels, the concentration was approximately 2 log lower than the exposition level after 24 h from the contamination, and then it decreased exponentially of 1 log every 24 h. Our findings suggest that in the experimental conditions tested *A. butzleri* is neither able to effectively grow in seawater nor bioaccumulate in mussels, at least in the free and cultivable form.

## 1. Introduction

The genus *Arcobacter* had become increasingly important in recent years because some of the species have been considered emergent food-borne enteropathogens worldwide [1]. *A. butzleri *is the most important and prevalent species of the genus: it has been classified as a serious hazard to human health by the International Commission on Microbiological Specifications for Foods [2] and a significant zoonotic pathogen [3]. In Europe, for example, *A. butzleri *has been reported as the fourth most common *Campylobacter*-like organism recovered from stools of patients with diarrhoea in Belgium and France [4, 5]. Moreover, in Italy an outbreak of *A. butzleri *affected 10 children, with such severe symptoms to require hospitalization [6]. This species has also been reported to be the etiological agent of traveller's diarrhoea acquired by U.S. and European travellers to Mexico, Guatemala, and India, with a prevalence of 8% [7]. *A. butzleri *has also been frequently isolated from the intestinal tracts and faecal samples of different farm animals and, after excretion, this microorganism can persist in the environment [8]. In this regard, the aquatic environment is a receptacle of agricultural and urban waste water effluents and *A. butzleri *has been detected in coastal areas [8, 9]. Consequently, bivalve molluscs can concentrate this microorganism from contaminated water during their filter-feeding activities [10, 11]. Despite this, the behaviour of this pathogen in the marine environment and how it bioaccumulates in shellfish have not been investigated, although the public health risk associated with the consumption of shellfish contaminated with other human enteropathogens is well documented [12]. Based on these considerations, the aim of this work was to evaluate, at the laboratory scale, the ability of *Arcobacter* to grow in seawater and bioaccumulate in mussels, in order to gain preliminary information on humans risk related to the presence of *A. butzleri *in the marine environment.

## 2. Material and Methods

### 2.1. Samples

Blue mussels (*Mytilus galloprovincialis*) of average size (5 ± 7 cm length) were collected from an authorized harvesting area of the Central Adriatic sea (Italy), classified as category A (postharvested treatment for human consumption is not required), suitably chosen because negative for the research of *Arcobacter* performed monthly, in the six months previous to the execution of the described experiments. Mussels were transported to the laboratory in refrigerated containers (4°C), where they were inspected. Dead or damaged specimens were eliminated. After roughly removing from the organisms the most of mud, encrustations, epiphytes, and epizoa [13], mussels were divided into aliquots of 20 elements each and closed in special mesh bags. 

### 2.2. Bacterial Strains

The strain used for the bioaccumulation experiments was *A. butzleri *LMG 10828^T^. This was maintained in Arcobacter broth (Oxoid) and stored at 3°C ± 2°C for up to a week. 

For the experiments *A. butzleri* grown in Tryptone Soy Broth (Oxoid) at 30°C for 48 h was centrifugated at 5000 ×g for 20 min at 4°C. Pellet was resuspended in phosphate buffer saline (PBS, 10% w/v), adjusted to the concentration of 5 × 10^9^ CFU/mL after optical measurement (1 × 10^8^ CFU/mL gave ca. 0.1 OD600 nm), and then stored at 3°C ± 2°C for up to 1 hour before use. 

### 2.3. Acclimation of the Mussels

Prior to each experiment, 20 mussels were evenly distributed into each of four tanks containing 20 liters of artificial seawater (Instant Ocean Aquarium Systems), approximately 1 L of water per mussel. The conditions in the tanks were the following: salinity, 2.8–3.3%; temperature, 16–18°C, and constant aeration [13]. At time 0 h, a ration of Instant Algae Shellfish Diet 1800 (Reed Mariculture, Campbell, CA), consisting of four inactivated algae (*Isochrysis*, *Pavlova*, *Thalassiosira weissflogii*, and *Tetraselmis*), was added to each tank, at the rate of 1 × 10^6^ algae/mL of seawater [14]. Mussels were first allowed to adapt for 24 h [13, 14]; then, after visually assessing that they were alive, the four tanks were randomly assigned to the negative-control (no *A. butzleri *added) or one of the three test groups. Mussel-feeding was repeated at the same rate at 24 h and then reduced to 5 × 10^5^, 5 × 10^4^, 5 × 10^3^ algae/mL of water at 48, 72, and 96 h, respectively, since a portion of the organisms had been removed for daily testing [14].

### 2.4. Bioaccumulation Experiments

200 mL of PBS containing about 5 × 10^8^, 5 × 10^6^, and 5 × 10^4^ CFU/mL of *A. butzleri *LMG 10828^T^ were inoculated in the tank of the first, second, and third test groups, respectively, to achieve the final concentrations of about 5 × 10^6^ CFU/mL, 5 × 10^4^ CFU/mL, and 5 × 10^2^ CFU/mL. Immediately after the experimental contamination, 10 mL of water from each tank were collected, in order to determine the concentration of *A. butzleri*. Then, the enumeration of *A. butzleri* was performed each day for all the test groups and the negative control from both water and mussels, for up to 96 h. Three other test groups represented by tanks containing 20 L of artificial seawater without mussels and aeration were contaminated and daily analyzed in the same manner as the water in the experiments with mussels. 

### 2.5. Preparation of the Samples

After having carefully cleaned externally the organisms and eliminated the residual traces of mud, encrustations, epiphytes, and epizoa, mussels were aseptically prepared for analysis in accordance with a standard procedure [15]. For each sample, constituted by 5 elements [14], 10 g of digestive tissues (DT) and 10 g of the remaining part of the body, including the liquor (BL), were collected separately, diluted 1 : 10 with 0.1% peptone (w/v) 0.85% salt (w/v) water, and homogenized in a blender. 

### 2.6. Enumeration of *A. butzleri*

Both water and mussel homogenates were subjected to serial dilutions using the same buffer to as high as 10^−6^ and *A. butzleri *was enumerated using a conventional pour plate method, inoculating 10 mL of each dilution on three plates (3 mL, 3 mL, and 4 mL for each plate) of Trypticase Soy Agar (Oxoid) plus 5% sheep blood [8, 16]. After incubation at 30°C for 48 h, typical colonies were counted. 

In this medium typical colonies were small, colorless or beige to off-white translucent, and of 1 to 4 mm in diameter [8, 16]. Presumptive *Arcobacter* colonies were confirmed with Gram stain (Gram negative, curved, or slightly curved rod) and oxidase test (positive). The definitive identification of the species was performed by PCR of the *rpoB* gene [16]. Genotyping by ERIC-PCR [17] was also performed to verify that the isolates belonged to the type strain used for contamination.

### 2.7. Statistical Analysis

Three independent experiments were performed for mussels collected at different times from each of the test group and for artificial seawater from both the experiments with and without mussels. Each analysis was performed by analyzing water, DT, and BL for three times. For each test group mean values (*n* = 9) and standard deviations were calculated. The Student's *t*-test was used to compare the means of the bacterial counts obtained from DT and BL for each level of experimental contamination and a probability value (*P*) < 0.05 was regarded as statistically significant. 

## 3. Results and Discussion

**Figure 1:**
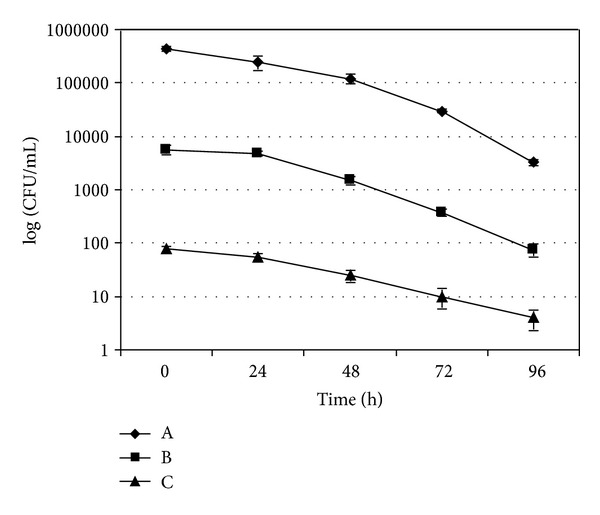
Comparison of level of *A. butzleri* LMG 10828^T^ in water without mussels at time 0 and at 24, 48, 72, and 96 h after contamination with about 5 × 10^6^ CFU/mL (A), 5 × 10^4^ CFU/mL (B), and 5 × 10^2^ CFU/mL (C). The error bars indicate the standard deviation of three independent experiments each performed in triplicate (*n* = 9).

**Figure 2:**
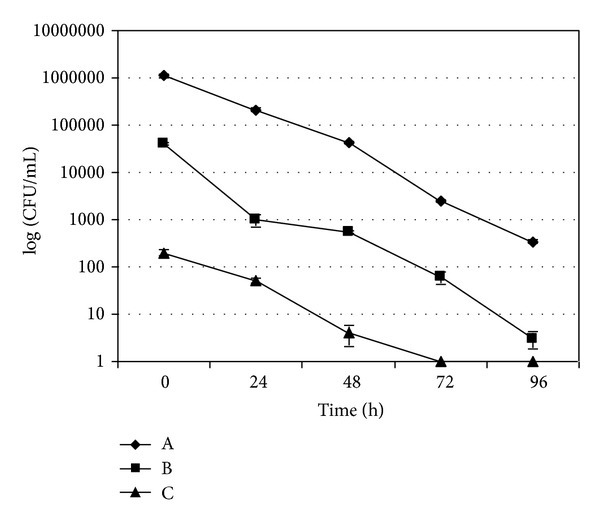
Comparison of level of *A. butzleri* LMG 10828^T^ in water with mussels at time 0 and at 24, 48, 72, and 96 h after contamination with about 5 × 10^6^ CFU/mL (A), 5 × 10^4^ CFU/mL (B), and 5 × 10^2^ CFU/mL (C). The error bars indicate the standard deviation of three independent experiments each performed in triplicate (*n* = 9).

**Figure 3:**
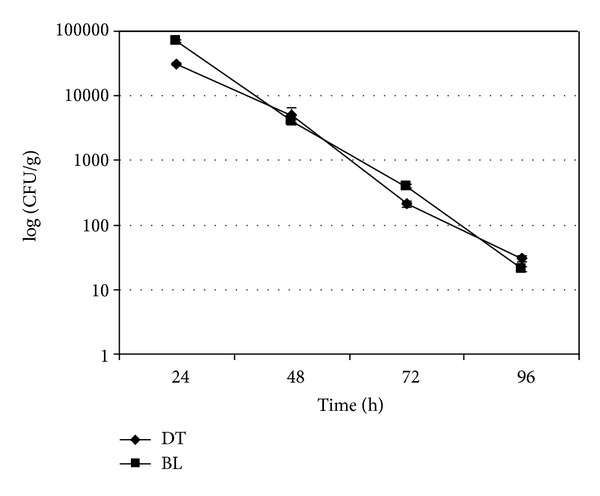
Comparison of level of *A. butzleri* LMG 10828^T^ in digestive tissue (DT) and in the remaining body and liquor (BL) of mussels at 24, 48, 72, and 96 h after water contamination with about 5 × 10^6^ CFU/mL. The error bars indicate the standard deviation of three independent experiments each performed in triplicate (*n* = 9).

**Figure 4:**
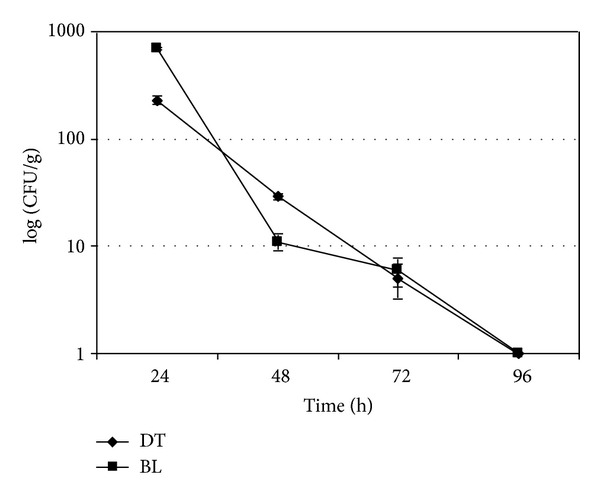
Comparison of level of *A. butzleri* LMG 10828^T^ in digestive tissue (DT) and in the remaining body and liquor (BL) of mussels at 24, 48, 72, and 96 h after water contamination with about 5 × 10^4^ CFU/mL. The error bars indicate the standard deviation of three independent experiments each performed in triplicate (*n* = 9).

In each experiment, the uncontaminated mussels used as negative controls always resulted negative for *A. butzleri* (data not shown). Microbiological trends of *A. butzleri* in experimentally contaminated mussels and water are shown in Figures 1–4. In the experiments without the mussels the concentration of *A. butzleri *in water decreased less than 0.5 log every 24 h in the first 48 h, and then decreased of about 1 log every 24 hours (Figure 1). In the experiments with mussels, *A. butzleri *declined in water exponentially over time, with a reduction of about 1 log every 24 h (Figure 2). The greater reduction in the first 48 h of the loads of *A. butzleri* in the tests conducted in the water with mussels than in those without the mussels can be probably attributed to the presence of the animals and their capacity to filter the microorganism from the water. In the contamination experiments with about 5 × 10^2^ CFU/mL, *A. butzleri* was no more detectable after 72 h from the contamination in the water with mussels (Figure 2). Considering instead the microbiological trend of *A. butzleri* in mussels, for all levels of the experimental contamination tested, the counts obtained from DT were not statistically different from those obtained from BL (Figures 3 and 4). This suggests that, unlike other pathogens [18], for *A. butzleri* DT does not represent an elective tissue for bioaccumulation. After 24 h from the contamination, the concentration of *A. butzleri* in the mussels was approximately 2 log lower than the concentration in water at the time 0; then it decreased exponentially of about 1 log every 24 h (Figures 2, 3, and 4). In the bioaccumulation test with a level of water contamination of about 5 × 10^2^ CFU/mL, *A. butzleri* was not detectable in mussels at 24 h from contamination, remaining not detectable after 48, 72, and 96 h (data not shown). 

Fecally contaminated water and food products (especially poultry and red meat, milk, and shellfish, which had been often shown to be contaminated with arcobacters) have been suggested as the transmission routes for *A. butzleri* [1]. It was also hypothesized that clinical relevant *Arcobacter* species could be autochthonous to marine environments [9]. Previous studies reported a high prevalence of this pathogen in faeces of livestock animals [19] and in farm effluents [20] and this finding could indicate that those are the real sources of surface water contamination. Moreover, *A. butzleri* is significantly more prevalent in water that is fecally contaminated than in water that is not [8]. Based on these pieces of evidence, we also believe that this species arrives in seawater through polluted fresh water. Moreover, our results, obtained at laboratory scale, suggest that *A. butzleri* is not able to effectively grow in seawater in free and cultivable form. Of course we can not exclude the possibility that this organism in marine environment may instead find suitable conditions for the survival and rooting, adhering to zooplankton as previously suggested [9, 21]. The observed differences in clearance of bioaccumulated bacteria and viruses raise questions about potential interactions between shellfish and human pathogens [18]. For Norovirus, for example, bioaccumulation specifically occurred in digestive tissues by binding to specific ligands [18]. For other enteropathogens as *Salmonella *their capability of long-term persistence within shellfish through an unusual mechanism of colonization has been demonstrated [22, 23]. Although species like *A. mytili *and *A. molluscorum *can be commonly found in mussels [1, 16], our trends of the microbial counts of *A. butzleri *in both DT and BL suggest that this species, or at least the type strain, is neither able to effectively bioaccumulate in mussels nor to grow at cultivable form within these organisms for over than 96 hours after the experimental contamination.

## 4. Conclusions

The significance of *A. butzleri *as a human pathogen is not fully understood at the present, although, considering its isolation in cases of human and animal illness and from foods of animal origin, it may certainly be added to the ranks of emerging food-borne pathogens. For this reason, it is necessary to acquire more information on its epidemiology, occurrence in food, and survival strategies in the aquatic environment. 

We are aware of the limitations of this study, where only the type strain, that is not of marine origin, has been tested. Furthermore *in vitro* experiments may not represent what occurs in the natural environment. For these reasons, it is our intention to continue investigating this specific field, to get supplementary data by performing experiments with different shellfish and strains of *A. butzleri*.

To our knowledge, this is the first study which tested, at a laboratory scale, the performances of *A. butzleri* to grow in seawater and bioaccumulate in mussels, providing preliminary information on risk linked to the presence of this pathogen in marine organisms which are also food for humans.
